# Human Interaction Classification in Sliding Video Windows Using Skeleton Data Tracking and Feature Extraction †

**DOI:** 10.3390/s23146279

**Published:** 2023-07-10

**Authors:** Sebastian Puchała, Włodzimierz Kasprzak, Paweł Piwowarski

**Affiliations:** Institute of Control and Computation Engineering, Warsaw University of Technology, ul. Nowowiejska 15/19, 00-665 Warszawa, Poland

**Keywords:** human interaction videos, LSTM, preliminary skeleton features, skeleton tracking, sliding window, many-interaction videos

## Abstract

A “long short-term memory” (LSTM)-based human activity classifier is presented for skeleton data estimated in video frames. A strong feature engineering step precedes the deep neural network processing. The video was analyzed in short-time chunks created by a sliding window. A fixed number of video frames was selected for every chunk and human skeletons were estimated using dedicated software, such as OpenPose or HRNet. The skeleton data for a given window were collected, analyzed, and eventually corrected. A knowledge-aware feature extraction from the corrected skeletons was performed. A deep network model was trained and applied for two-person interaction classification. Three network architectures were developed—single-, double- and triple-channel LSTM networks—and were experimentally evaluated on the interaction subset of the ”NTU RGB+D” data set. The most efficient model achieved an interaction classification accuracy of 96%. This performance was compared with the best reported solutions for this set, based on “adaptive graph convolutional networks” (AGCN) and “3D convolutional networks” (e.g., OpenConv3D). The sliding-window strategy was cross-validated on the ”UT-Interaction” data set, containing long video clips with many changing interactions. We concluded that a two-step approach to skeleton-based human activity classification (a skeleton feature engineering step followed by a deep neural network model) represents a practical tradeoff between accuracy and computational complexity, due to an early correction of imperfect skeleton data and a knowledge-aware extraction of relational features from the skeletons.

## 1. Introduction

Human activity recognition in image sequences and video has lately been a hot research topic in the computer vision, multimedia, and machine learning communities. Two-person interactions constitute a specific category of human activities. Currently, the best performing solutions are based on deep learning techniques, in particular on deep neural networks (DNN) such as CNN (convolutional neural networks), GCN (graph convolutional networks, or LSTM (long short-term memory networks) [1,2,3,4]. Practical applications of related technology are expected in video surveillance, robotics, or content-based video filtering.

Human activity recognition in video can be divided into two main categories: applying the activity recognition method directly to video data [5] or first performing a human pose estimation (i.e., skeleton detection) in every frame of the sequence [6]. Nowadays, 2-dimensional (2D) or 3-dimensional (3D) human skeleton representations of human-populated image regions are generated sufficiently reliably, even with the support of specialized devices, such as the Microsoft Kinect. Some popular solutions to human skeleton estimation (i.e., detection and localization) in images can be mentioned: OpenPose [7], DeepPose [8], and DeeperCut [9]. There are three fundamental architectures, which have been employed as backbone architecture for human pose estimation research: AlexNet (e.g., in the DeepPose model), Visual Geometry Group network (VGG) (e.g., in OpenPose), and Residual Neural Network (ResNet) (e.g., in DeeperCut). In early solutions, hand-designed features, such as edges, contours, Scale-Invariant Feature Transform (SIFT), and Histogram of Oriented Gradients (HOG), have usually been used for the detection and localization of human body parts or key points in the image [10]. More recently, deep neural network-based solutions were successfully proposed [4], as they have the capability to automatically learn rich semantic and discriminative features. Initially, Multi-layer Perceptrons (MLP) and LSTM models were explored but, currently, Convolutional Neural Networks (CNN) and Graph CNNs [11] dominate the research. CNNs can learn both spatial and temporal information from signals and can effectively model scale-invariant features as well.

In a recent work [12], we proposed knowledge-aware feature extraction from skeleton data. As relational features are mostly created from skeletons, this allowed us to focus subsequently on the temporal aspect and to use a single-channel LSTM network instead of the often-proposed CNNs and GCNs. In this work, two novel issues were studied. First, various multi-stream networks (single-, double- and triple-channel networks) with LSTM layers were proposed, performing feature processing and classification. This led to new findings and increased the classification accuracy. The second issue was the implementation of a sliding window technique to process longer time video clips, containing many different activities. This will allow the development of different strategies for the overall classification of a video clip.

There are four remaining sections of this work. Section 2 describes recent approaches to human-activity classification. Our solution is presented in Section 3. In Section 4, experiments are described that verify different network architectures when processing three different feature sets. All models were learned and evaluated on the interaction subset of the NTU RGB+D data set [1]. The models learned on the main data set and the sliding window strategy were also cross-validated on the UT-Interaction data set [13]. Finally, in Section 5, we summarize our results.

## 2. Related Work

The recognition of human activities in video is a hot research topic in the last fifteen years. Typically, human activity recognition in images and video requires first a detection of human body parts or key-points of a human skeleton. The skeleton-based methods compensate for some of the drawbacks of vision-based methods, such as assuring the privacy of persons and reducing the scene lightness sensitivity.

Most of the research is based on the use of artificial neural networks. However, more classical approaches have also been tried, such as the SVM (e.g., [14,15]). Yan et al. [16] used multiple features, such as a “bag of interest points” and a “histogram of interest point locations”, to represent human actions. They proposed a combination of classifiers in which AdaBoost and “sparse representation” were used as basic algorithms. In the work of Vemulapalli et al. [17], human actions were modeled as curves in a Lie group of Euclidean distances. The classification process uses a combination of dynamic time warping, Fourier temporal pyramid representation, and linear “support vector machine” (SVM).

Thanks to higher quality results, artificial neural networks are replacing other methods. Thus, the most recently conducted research in human activity classification differs only in terms of the proposed network architecture. Networks based on the LSTM architecture or a modification of this architecture (a ST-LSTM network with trust gates) were proposed by Liu et al. [18] and Shahroudy et al. [1]. They introduced so-called “Trust Gates” for controlling the content of an LSTM cell and designed an LSTM network capable of capturing spatial and temporal dependencies at the same time (denoted as ST-LSTM). The task performed by the gates is to assess the reliability of the obtained joint positions based on the temporal and spatial context. This context was based on the position of the examined junction in the previous moment (temporal context) and the position of the previously studied junction in the present moment (spatial context). This behavior is intended to help network memory cells assess which locations should not be remembered and which ones should be kept in memory. The authors also drew attention to the importance of capturing default spatial dependencies already in the skeleton data. They experimented with different joint’s set-to-sequence mappings. For example, they mapped the skeleton data into a tree representation, duplicating joints when necessary to keep spatial neighborhood relation and performed a tree traversal to obtain a sequence of joints. Such an enhancement of the input data allowed an increase of the classification accuracy by several percent.

The work [19] introduced the idea of applying convolutional filters to pseudo-images in the context of action classification. A pseudo-image is a map (a 2D matrix) of feature vectors from successive time points, aligned along the time axis. Thanks to these two dimensions, the convolutional filters find local relationships of a combined time–space nature. Liang et al. [20] extended this idea to a multi-stream network with three stages. They used three types of features, extracted from the skeleton data: positions of joints, motions of joints, and orientations of line segments between joints. Every feature type was processed independently in its own stream but after every stage the results were exchanged between streams.

Graph convolutional networks are currently considered a natural approach to the action (and interaction) recognition problem. They are able to achieve high quality results with only modest requirements of computational resources [21,22].

One of the best performances on the NTU RGB+D interaction data set is reported by the work [3]. Its main contribution is a powerful two-stream network with three-stages, called “Interaction Relational Network” (IRN), the input to which is the basic relations between the joints of two interacting persons tracked over the length of image sequence and then makes further encoding, decoding, and an LSTM-based final classification. In our view, the most important contribution is to the initial extraction of the well-structured preparation of pair-wise input relation that contains both distance and motion information between joints, where the first stream processes within-a-person relations, while the second one —processes between-person relations. The use of final LSTM represents a high-quality model, called the IRN-LSTM network. It leads to the processing of a dense frame sequence, so all frames of the video clip can be processed. Instead of an LSTM, in ordinary versions of the IRN network, a simple densely connected classifier is used and a sparse sequence of frames is processed.

Another recent development is the pre-processing of the skeleton data to extract different types of information (e.g., information on joints and bones and their relations in space and time). Such data streams are first separately processed by so called multi-stream neural networks and later fused to a result. Examples of such solutions are the “Two-Stream Adaptive Graph Convolutional Network” (2S-AGCN) and the “Multistream Adaptive Graph Convolutional Network” (AAGCN), proposed by Shi et al. [23,24].

The current best results for small-size networks were reported by Zhu et al. [25], where two new modules were proposed for a baseline 2S-AGCN network. The first module extends the idea of modelling relational links between two skeletons by a spatial-temporal graph to a “Relational Adjacency Matrix (RAM)”. The second novelty is a processing module, called “Dyadic Relational Graph Convolution Block”, which combines the RAM with spatial graph convolution and temporal convolution to generate new spatial-temporal features.

Very recently, exceptionally high performance was reported when using networks with 3D convolutional layers, applied to data sensors that constitute skeleton “heatmaps” (i.e., preprocessed image data) [26]. The approach, called PoseConv3D, can even be topped when fused with the processing of ordinary RGB-data streams [27]. Obviously, this requires the creation of a heavy network and produces high computational load.

From the analysis of the recent successful solutions, we drew three main conclusions and motivation for our research work:

1. Using many streams of skeleton data (i.e., joints, branches, spatial and temporal interrelations) was proved to provide essential and meaningful information for activity classification (e.g., interaction relational networks, two- and multi-stream DNN architectures);
2. The use of light-weight solutions is preferred in practice, achieved by using graph CNNs combined with ordinary CNNs and using CNNs with 2-D kernels instead of 3-D CNNs, although heavy-weight solutions, such as 3D CNNs, are topping the performance rankings;
3. In practice, a video clip (or a particular time-window), apparently containing a human action or interaction, is reduced to a sparse frame sequence, although using all the available frames improves the performance.

## 3. The Approach

### 3.1. Structure

**Figure 1:**
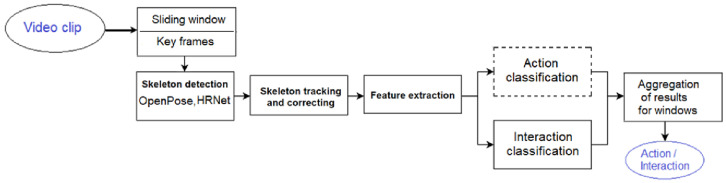
General structure of our approach.

A video clip may contain many activities of the same or different persons. Thus, the video is analyzed in short-time chunks created by a sliding window. A fixed number of video frames is selected from every data chunk for further analysis. As shown in Figure 1, the proposed solution consists of the following main processing stages:

1. Sliding window and key-frame selection: a fixed number of frames, selected from a time-window of frames, is assumed to be analyzed further;
2. Skeleton detection and estimation: a pose estimator (e.g., the OpenPose net [7]) is applied to detect and localize human skeletons and their 2D joints in every RGB image (selected video frame) of an image sequence;
3. Skeleton tracking and correcting: two “main” skeletons are tracked in the image sequence; low certain joints or missing joints are replaced by interpolated data;
4. Feature extraction: features are created from the two streams of joints; we studied three types of relational features, besides the raw skeleton data;
5. Neural network models: alternative LSTM-based models are trained and applied for action- and interaction classification (please note, that the topic of this paper is limited to the interaction classification case).

### 3.2. Sliding Window and Key-Frame Selection

**Figure 2:**
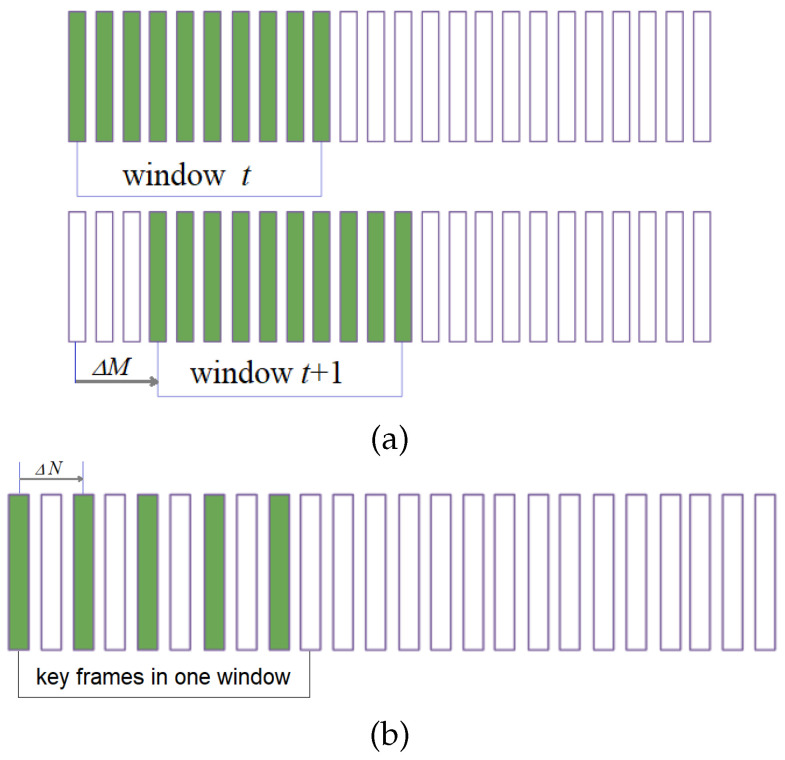
Sparse frame sequence created (**a**) by current location of a sliding window, with adjustable size and window interlace ratio; and (**b**) selection of a fixed number of frames.

A basic design question is the generation of image (frame) sequences from a video clip. Videos can be of different lengths; the duration of actions and frame rates can differ. Theoretically, Recursive Neural Networks (RNN) can be adopted to operate on a variable-length input. However, this is not recommended (such networks are more difficult to learn). Thus, we decided to use image sequences of fixed length, extracted by a sliding window (Figure 2a). With this approach, many sub-sequences may be created for an input video in the testing and active work mode. It must be noted that, for training a neural network model, short-time video clips are used, converted to single windows, as a single reference label is assigned to every sample clip.

The key issue is to choose the right length of the sliding window. If a short-time video clip is processed, which contains one activity type only, the window should cover nearly the entire clip. When a longer-time video may contain many activity instances, the window should be able to cover a single activity only. We decided to operate with a window length of 2.133 s, which corresponds to a number *M* of 64 frames. As the labeled training samples with single interactions are typically of length 2.5–3 s, the selected window length should satisfy both above requirements.

The number of key frames *N* in a window must be consistent with the input size of the trained or applied neural network model. In the literature dedicated to this topic, typically *N* is chosen in the range from 8 to 32 or all frames of a video clip are considered (limited only by assumed window size). By choosing N=32 keyframes, we achieved a fair comparison with recent results of other researchers, using the same amount of information, and also had a chance to process the video in real time. With experiments, we confirmed that, with a growing number of keyframes, the classification accuracy is steadily improving.

After fixing the window length and the number of key frames in the sliding window, another two parameters must be selected: the interlace ratio of (or delay ΔM between) two consecutive windows and the frame rate (or delay ΔN between consecutive key frames) in a window (Figure 2b).

### 3.3. Skeleton Detection and Estimation

In the paper [7], a multi-person 2D pose estimation architecture was proposed based on “Part Affinity Fields” (PAFs). The work introduced an explicit nonparametric representation of the key point association, which encodes both position and orientation of the human limbs. The designed architecture can learn both human key point detection and association using heatmaps of human key-points and part affinity fields, respectively. It iteratively predicts part affinity fields and part detection confidence maps. The part affinity fields encode part-to-part association including part locations and orientations. In the iterative architecture, both PAFs and confidence maps are iteratively refined over successive stages with intermediate supervision at each stage. Subsequently, a greedy parsing algorithm was employed to effectively parse human poses. The work ended up releasing the OpenPose library, the first real-time system for multi-person 2D pose estimation.

In our research, we alternatively used OpenPose or HRNet. The core block of OpenPose, the “body_25 model” containing 25 characteristic points (joints) located in the image. Every joint $o_{i}$, estimated by the OpenPose system, is described in the following format: $o_{i}=\left(x_{i},y_{i},c_{i}\right)$, where $\left(x_{i},y_{i}\right)$ are absolute pixel coordinates of junction *i* in the image and $c_{i}$ is the certainty of joint detection—a value from the range [0, 1].

Thus, for each frame *t* and skeleton *p*, we get a vector of raw characteristics $v_{p}^{t}$, which has 75 elements.

### 3.4. Skeleton Tracking and Correcting

**Figure 3:**
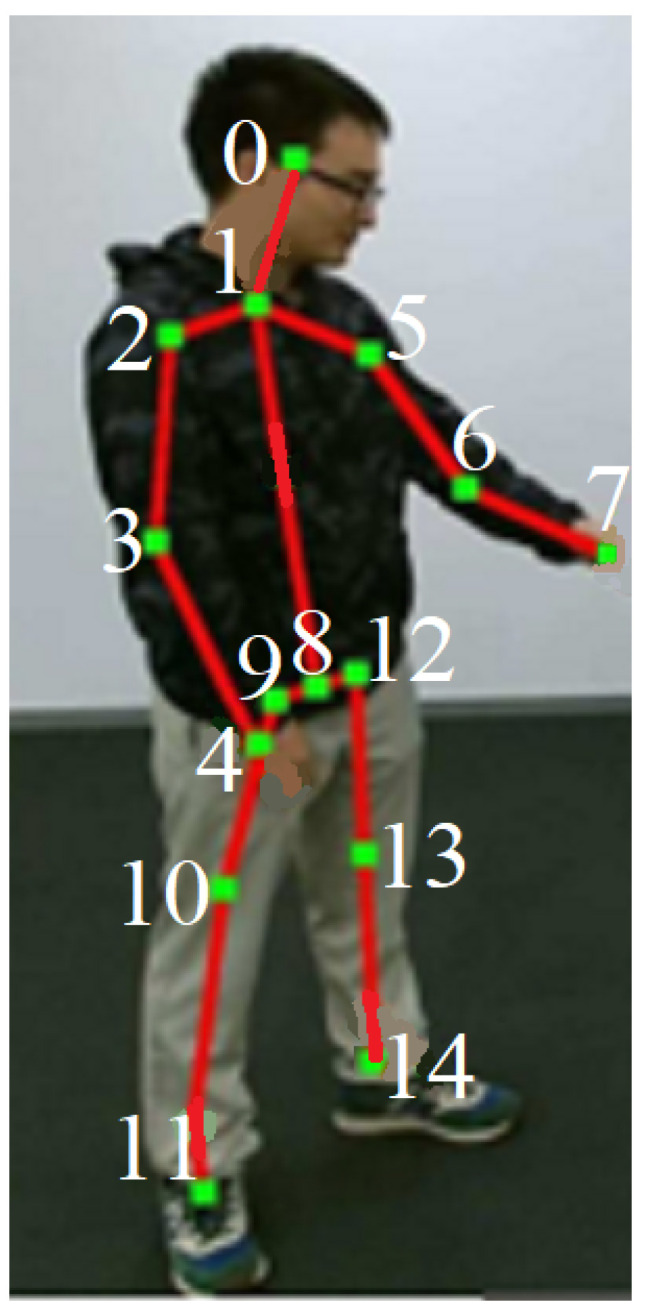
The 15 reliable joints (marked from 0 to 14) out of 25 of the OpenPose’s “body_25” skeleton model with the size normalization distance $o_{1}⌢o_{8}$.

In cases where more than two skeletons in an image are returned by OpenPose/HRNet, the two largest skeletons are selected first and next they are tracked in the remaining frames. We focused on the first 15 joints of every skeleton—a conclusion from an statistical evaluation of the detected skeletons (Figure 3).

We also canceled some joints data that are uncertain. Those values, whose certainty value is $c_{i}<0.3$, were removed and replaced by a special mark representing “not a value”.

Finally, the absolute image coordinates were transformed into relative coordinates by dividing them by the corresponding image size.

The location data for joints received from OpenPose are not always perfect. It happens that some joints are not detected, while some others are detected with low certainty, and we removed them. Fortunately, due to the sequential nature of the available data, a variety of techniques can be used to fill these gaps. Let $v_{i}$ be a series of *N* positions $o_{i}^{t}$ of joint *i* in time: $v_{i}=\left[o_{i}^{1},o_{i}^{2},...,o_{i}^{N}\right]$. The following techniques were applied to improve the quality of skeleton data:

1. Problem: one position $o_{i}^{t}$ is missed; solution: taking the average of neighbors across time $o_{i}^{t}=0.5\left(o_{i}^{t-1}+o_{i}^{t+1}\right)$′;
2. Problem: $o_{i}$ is missed *k* consecutive times, i.e., from *t* to t+k−1; solution: taking an interpolation of values $o_{i}^{t-1}$ and $o_{i}^{t+k}$;
3. Problem: $o_{i}$ is missed first *k* times; solution: set first *k* values of $o_{i}^{t}$ to $o_{i}^{k+1}$;
4. Problem: $o_{i}$ is missed last *k* times; solution: set last *k* values $o_{i}^{t}$ to $o_{i}^{N-k}$;
5. Problem: $o_{i}$ is completely missed; solution: set it by default, relative to known joints.

The result of tracking (up to) two sets of skeleton joints in *N* frames can be represented as a 2D map of N×15×2 entries:

(1) $$V^{N}=\left[\begin{array}{ccc} v_{p1}^{1} & ⌣ & v_{p2}^{1}\\ v_{p1}^{2} & ⌣ & v_{p2}^{2}\\ ... & ⌣ & ...\\ v_{p1}^{N} & ⌣ & v_{p2}^{N} \end{array}\right]$$

where every $v_{p}^{i}=\left[o_{1}^{i},o_{2}^{i},...,o_{1}5^{i}\right]$ is a vector of 15 joints, represented by location coordinates, of skeleton *p* in frame *i*.

### 3.5. Feature Extraction

Unfortunately, such a strict representation of junction data, as in Equation (1) has obvious disadvantages—the data are not invariant with respect to the position in the image and do not explicitly represent relationships between both skeletons. First, the coordinates of the joints may randomly change but still represent the same semantic meaning (i.e., an action stage). The second problem is that the distance of points during interaction depends on the scale of the presentation of the scene in the image and the size of people. Thirdly, the point representation does not explicitly model other important relationships between silhouettes such as relative orientation and movement. Of course, a deep network would also be able to learn such dependencies but then we unnecessarily lose computing resources and deteriorate the quality of predictions for learning data transformations, which can be easily performed analytically. Therefore, three types of mutual representation of both skeletons were developed, which reduce the disadvantages of the “raw” representation of joints:

1. Limb-angle features—in the further part of the work, also called “LA features”;
2. Polar dense features (PD);
3. Polar sparse features (PS).

#### 3.5.1. Size Normalization

The invariance of features with respect to the size of the skeleton in the image was obtained by normalizing the coordinates of the junction points with the section between the neck $o_{1}$ and the center of the hips $o_{8}$ (Figure 3). This distance is most often correctly detected by OpenPose. Secondly, it does not depend on the angle of human position in relation to the camera. The only exception is when the person’s spine is positioned along the depth axis of the camera system (this case does not occur in the data sets used). After calculating the length of the segment $o_{1}⌢o_{8}$, it becomes a normalization value for all other measured distances in the feature sets. This distance is measured only for the first person and both persons are normalized by it.

#### 3.5.2. LA Features

**Figure 4:**
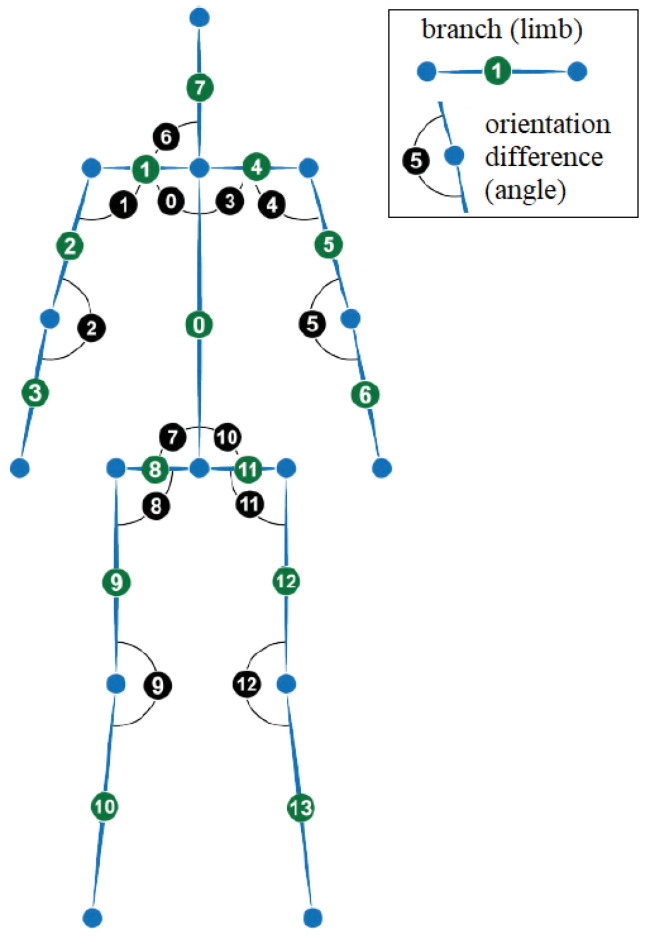
Illustration of the LA features: 14 line segments (called “limbs”) of a skeleton and 13 orientation changes between neighbor segments.

For every skeleton *a* and *b* the following are obtained (Figure 4): the lengths of 14 line segments (called “limbs”) $f_{a}$, $f_{b}$ (distances between two neighbor joints) and 13 orientation changes (angles) $r_{a}$, $r_{b}$ between two neighbor segments (Figure 4). Additionally, distances d(j) between pairs of corresponding joints (the same index *j*) of two skeletons *a* and *b* are also considered (15 distances).

Thus, for every frame, 69 features are defined, =(14+13)·2+15. The N·69 features are split into two maps, one for each skeleton, $F_{a}^{N}$ and $F_{b}^{N}$, with common part (15 distances $d{\left(j\right)}^{t}$ for every frame *t*) provided in both maps:

(2) $$F_{a}^{N}=\left[\begin{array}{ccccc} f_{a}^{1} & ⌣ & r_{a}^{1} & ⌣ & d^{1}\\ f_{a}^{2} & ⌣ & r_{a}^{2} & ⌣ & d^{2}\\ ... & ⌣ & ... & ⌣ & ...\\ f_{a}^{N} & ⌣ & r_{a}^{N} & ⌣ & d^{N} \end{array}\right]$$

(3) $$F_{b}^{N}=\left[\begin{array}{ccccc} f_{b}^{1} & ⌣ & r_{b}^{1} & ⌣ & d^{1}\\ f_{b}^{2} & ⌣ & r_{b}^{2} & ⌣ & d^{2}\\ ... & ⌣ & ... & ⌣ & ...\\ f_{b}^{N} & ⌣ & r_{b}^{N} & ⌣ & d^{N} \end{array}\right]$$

#### 3.5.3. PD Features

**Figure 5:**
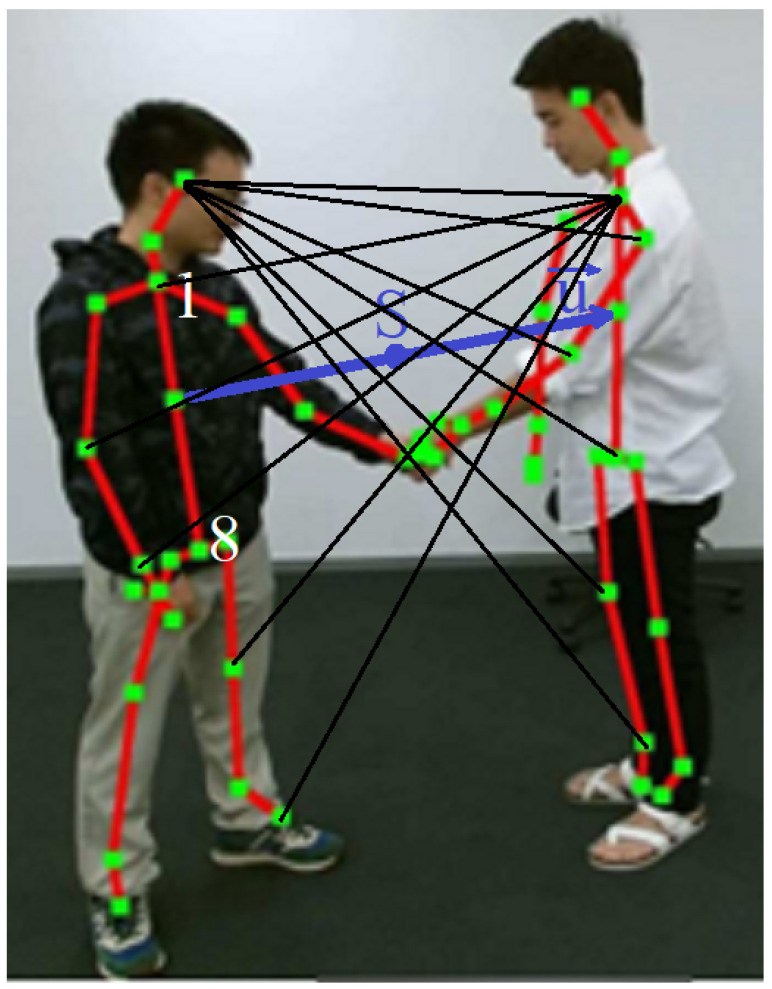
Illustration of the polar dense (PD) features: vectors between every pair of joints from different skeletons are computed and their lengths and orientations are normalized with respect to the vector **u**, drawn between centers of spinal segments (between joints 1 and 8) of both skeletons; S is the center point of vector **u**.

We define a vector u between the center points of the $o_{1}⌣o_{8}$ segments of both skeletons (Figure 5). This vector will be used to normalize the distances between joints of different skeletons and to make relative orientation of lines connecting the joints of different skeletons. The PD feature set includes vectors connecting every joint of first skeleton (a) with every joint of second skeleton (b) and vice versa—skeleton 2 with skeleton 1 (Figure 5). Every vector is represented in polar form by its magnitude $q_{a,j}$, $q_{b,j}$ (normalized by the distance of u and by its relative orientation $r_{a,j}$, $r_{b,j}$ (relative to the orientation of vector u). Thus, for every frame, there are 900 features defined (=225 (vector magnitudes) +225 (orientations) ·2. The N·900 features are split in two maps, $Q_{a}^{N}$ and $Q_{b}^{N}$, one for each skeleton:

(4) $$Q_{a}^{N}=\left[\begin{array}{ccc} q_{a}^{1} & ⌣ & r_{a}^{1}\\ q_{a}^{2} & ⌣ & r_{a}^{2}\\ ... & ⌣ & ...\\ q_{a}^{N} & ⌣ & r_{a}^{N} \end{array}\right]$$

(5) $$Q_{b}^{N}=\left[\begin{array}{ccc} q_{b}^{1} & ⌣ & r_{b}^{1}\\ q_{b}^{2} & ⌣ & r_{b}^{2}\\ ... & ⌣ & ...\\ q_{b}^{N} & ⌣ & r_{b}^{N} \end{array}\right]$$

#### 3.5.4. PS Features

**Figure 6:**
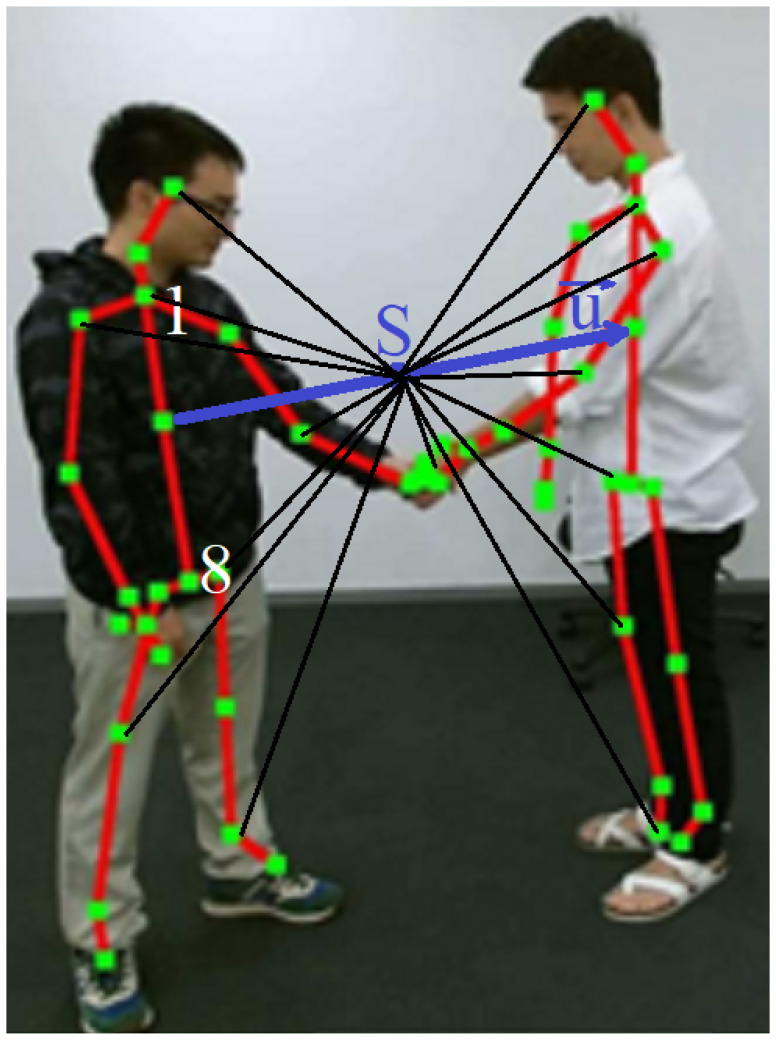
Illustration of the polar sparse (PS) features: vectors between center point *S* of vector **u** and every skeleton joint are computed and their lengths and orientations are normalized with respect to vector **u**, which connects the centers of spinal segments (between joints 1 and 8) of every skeleton.

Let us define the center point *S* of vector u (Figure 5). Now, 15 vectors are defined for every skeleton. Every vector connects the point *S* with a joint of skeleton 1 or 2 (Figure 6). Again, as for PD features, every vector is represented in polar form by two features—normalized magnitude $h_{a,j}$, $h_{b,j}$ and relative orientation $r_{a,j}$, $r_{b,j}$ (both magnitude and orientation are normalized with respect to u). Thus, for every frame there are 60 features defined only (=(15 + 15)·2). The N·60 features are split into two maps, $H_{a}^{N}$ and $H_{b}^{N}$, one for each skeleton:

(6) $$H_{a}^{N}=\left[\begin{array}{ccc} h_{a}^{1} & ⌣ & r_{a}^{1}\\ h_{a}^{2} & ⌣ & r_{a}^{2}\\ ... & ⌣ & ...\\ h_{a}^{N} & ⌣ & r_{a}^{N} \end{array}\right]$$

(7) $$H_{b}^{N}=\left[\begin{array}{ccc} h_{b}^{1} & ⌣ & r_{b}^{1}\\ h_{b}^{2} & ⌣ & r_{b}^{2}\\ ... & ⌣ & ...\\ h_{b}^{N} & ⌣ & r_{b}^{N} \end{array}\right]$$

### 3.6. LSTM Models

#### 3.6.1. Single Channel LSTM

**Figure 7:**
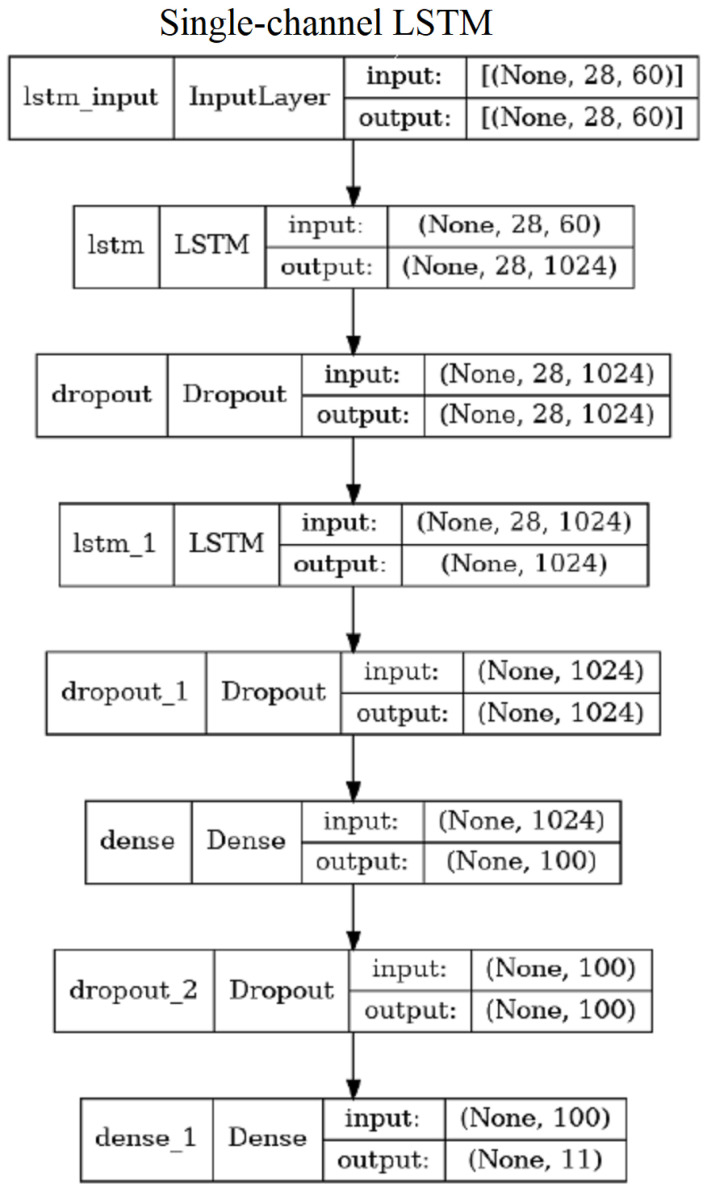
Illustration of the SC-LSTM-PS network—the three versions of SC-LSTM differ only in terms of the input layer size.

The “single channel” LSTM (SC-LSTM) has three versions corresponding to the three types of features (LA, PD or PS). Thus, we call them SC-LSTM-LA, SC-LSTM-PD, and SC-LSTM-PS, appropriately. We also considered a baseline feature version SC-LSTM-RAW, which processes the raw skeleton joints obtained by OpenPose. These versions differ by the input layer only, as there are different numbers of features considered. The network configuration consists of two LSTM layers, interleaved by two dropout layers and the final two dense layers (Figure 7). In the SC-LSTM-PS version, there are 3,359,931 trainable parameters.

#### 3.6.2. Double Channel LSTM

**Figure 8:**
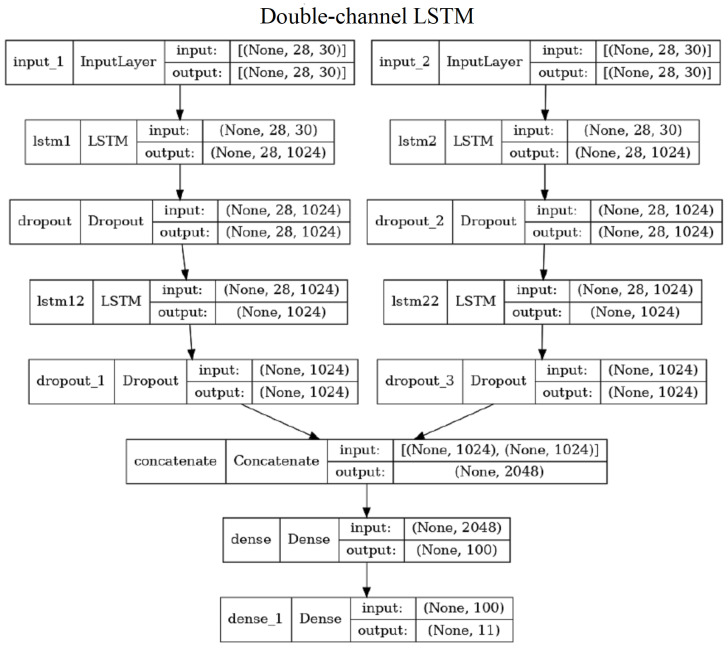
Illustration of the DC-LSTM-PS network—the two other versions of DC-LSTM differ only by the input layer size.

The “double channel” LSTM (DC-LSTM) has three versions corresponding to the three types of features (LA, PD, or PS). Thus, we call them DC-LSTM-LA, DC-LSTM-PD, and DC-LSTM-PS, appropriately. These versions differ in terms of the input layer only, as there are different numbers of features considered. The network configuration consists of two independent LSTM streams, a concatenation layer and two dense layers. Every LSTM stream has two LSTM layers interleaved by two dropout layers (Figure 8). The skeleton features are separated into two subsets, each corresponding to one skeleton. In the case of LA features, there is also a common part of both skeletons (15 distances between joints). This common data are added to the input of every stream. The DC-LSTM-PS network consists of 6,612,155 trainable parameters.

#### 3.6.3. Triple Channel LSTM

**Figure 9:**
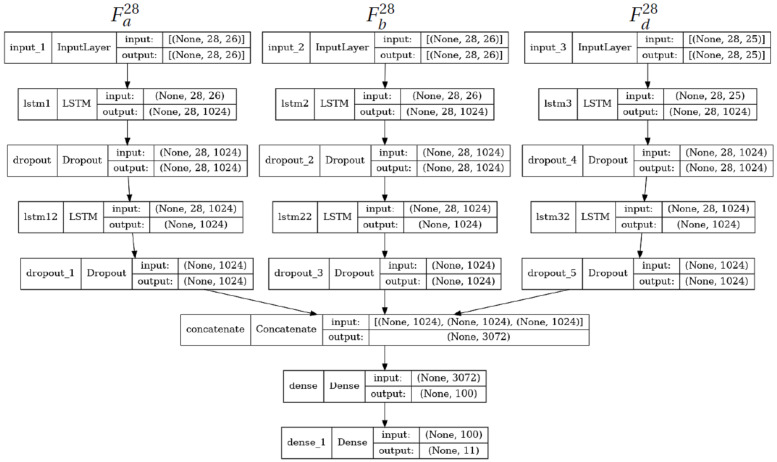
Architecture of the TC-LSTM-LA network.

The “triple channel” LSTM (DC-LSTM-LA) comes in one version only—for the LA features—as the other two features (PD and PS) have strictly two data streams only. The network configuration consists of three independent LSTM streams, a concatenation layer and two dense layers. Every LSTM stream has two LSTM layers interleaved by two dropout layers (Figure 9). Two of the LSTM streams process the feature subsets of every skeleton separately, while the third one processes the common feature subset (15 distances between joints). The TC-LSTM-LA network has 9,761,979 parameters.

## 4. Results

For evaluation of our approach and for performance comparison with other approaches to action and interaction classification, the “accuracy” metric and the class “confusion matrix” will be applied. “Accuracy” is the typical performance measure given in DNN-related publications [2] and is defined as a ratio of the correctly classified data to the total amount of classifications made by the model:

(8) $$Accuracy=\frac{Number\;of\;correct\;predictions}{Total\;number\;of\;predictions}.$$

Because of specific evaluation scenarios defined for the NTU RGB+D data set, called CS (cross-subject) and CV (cross-view), the test set is balanced with respect to classes and the class set is closed (i.e., all test samples belong to the known class set). Under these conditions, the “accuracy” value is equivalent to non-weighted (mean) average “recall”:

(9) $$Recall=\frac{1}{K}\sum_{i=1}^{K}\frac{TP_{i}}{TP_{i}+FN_{i}},$$

where *K* means the number of classes, $TP_{i}$—the number of true positives of class *i* samples, $FN_{i}$—the number of false negatives of class *i* samples.

### 4.1. Data Sets

To evaluate and test the trained classifiers, three data sets were used. The main data set on which our models were trained and evaluated was the interaction subset of the NTU RGB+D data set. It includes 11 two-person interactions of 40 actors: A50: punch/slap, A51: kicking, A52: pushing, A53: pat on back, A54: point finger, A55: hugging, A56: giving object, A57: touch pocket, A58: shaking hands, A59: walking towards, A60: walking apart.

In our experiments, the skeleton data of the NTU-RGB+D data set were already considered. There were 10.347 video clips in total, in which 7.334 videos were in the training set and remaining 3.013 videos were in the test set. No distinct validation subset was distinguished.

The NTU RGB-D data set allowed us to perform a cross-subject (person) (short: CS) or a cross-view (CV) evaluation. In the cross-subject setting, samples used for training show actions performed by half of the actors, while test samples show actions of remaining actors, i.e., videos of 20 persons were used for training and videos of the remaining 20 persons were used for testing. In the cross-view setting, samples recorded by two cameras were used for training, while samples recorded by the remaining camera were used for testing.

Each skeleton instance consists of 25 joints of 3D skeletons that apparently represent a single person. As our research objective was to analyze video data and to focus on only reliably detected joints, we used only the 2D information of the first 15 joints.

### 4.2. Verification on the NTU RGB+D Data Set

**Figure 10:**
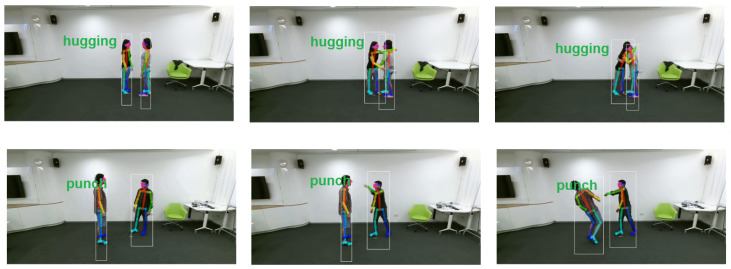
Illustration of properly classified interactions of hugging (**top row**) and punching (**bottom row**).

We trained and evaluated our eight models on the NTU RGB+D set, using only the 2D skeleton information, in both verification modes—CS (cross-subject) and CV (cross-view)—proposed by the authors of this data set. The training set was split into learning and test subsets—two thirds for learning and one third for validation/testing. CS means that actors in the training set are different than in the test set but data from all the camera views were included in both sets. CV means that two samples from camera views are included in the training set, while samples from the remaining camera view are in the test set. Some examples of proper interaction classification are shown in Figure 10.

**Figure 11:**
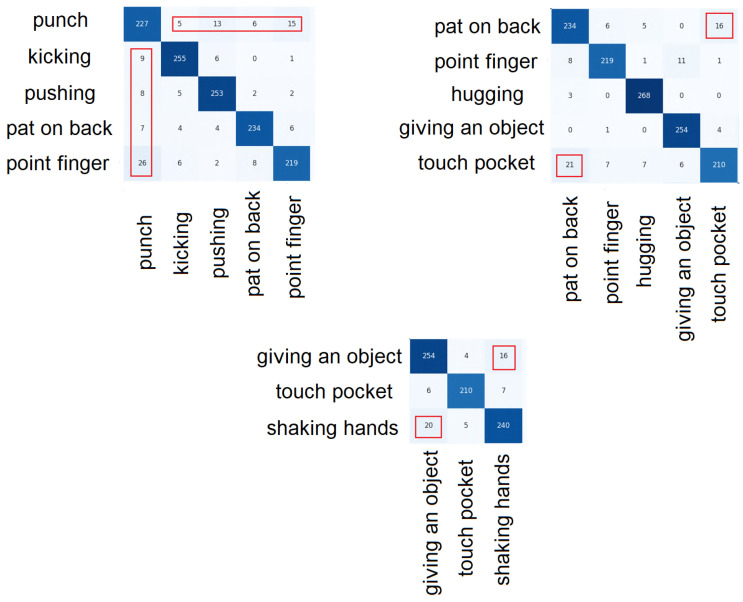
The most confusing cases of classification by the SC-LSTM-LA model.

Confusion matrices allow for accurate analysis of incorrect predictions of individual classes. In total, we prepared and analyzed 16 confusion matrices arrays (=8 models ×2 modes). Figure 11 shows fragments of a confusion matrix obtained for the SC-LSTM-LA model in the CS mode. We deliberately show results of an average performing model, so that any mistakes are more visible than in cases of better-performing models. The vast majority of class predictions are correct. The confused results are as follows:

- The punch class is confused with the finger pointing class—in both cases, a similar hand movement is made towards the other person;
- The class of pat on the back is confused with the class of touching a pocket—touching a pocket involves touching another person’s pocket in an interaction (a simulation of stealing a wallet), so the movement is close to pat someone on the back;
- The giving object class and the shaking hands class are very similar interactions—both involve the contact of the hand;
- The waking towards and waking apart classes are detected virtually flawlessly.

In addition, for three models, the per-class classification accuracy was computed (Table 1). We see exactly which classes cause the biggest problems. The worst-detected classes are: “punch”, “touch pockets” and “point finger”. However, all these errors almost disappear with the TC-LSTM-LA model, which detects all interaction classes at a similarly proper level.

The summary of results obtained by all the considered network architectures is given in Table 2. First, we clearly see the advantage of our feature engineering step, as all our models perform better with relational features than when using RAW skeleton data (SC-LSTM-RAW).

Consider now the effects of feature type and channel number. In the case of the SC-LSTM architecture, polar features (PD, PS) perform much better than the LA features. This was expected because the aim of using polar features is to more accurately represent interpersonal relationships. On the other hand, when the DC-LSTM architectures were compared, we see something completely different. The separation of channels for persons significantly improved the use of limb-angle features, while worsening the quality of polar features. In fact, the separation is very natural for LA features and the information related to every single person is independent of the other person. In the case of polar features, even when separated into two channels, they contain mutual information. This split of features gives no benefit and even causes a deterioration in quality. An interesting conclusion is also the similar level of performance of dense and sparse “polar” features, although their feature numbers are much different. The triple-channel configuration TC-LSTM-LA provides mixed results. It improves the accuracy of CS testing by 1.1% but deteriorates the CV testing by 1.2%.

We have chosen our three best performing models, SC-LSTM-PS, DC-LSTM-LA, and TC-LSTM-LA, for a comparison with other recent works.

### 4.3. Comparison Study

A complexity-to-quality tradeoff of our approach is demonstrated, when comparing it with other works referred in recent literature. A lot of works on two-person interaction classification have been evaluated on the NTU RGB+D interaction data set. In Table 3, we list some of the leading works with their accuracies given in referred works. A competitiveness of our three best models, regarding the criteria of quality and complexity, is observed. It must be noted that the top solutions use multi-data stream architectures. The PoseConv3D(J+L) solution is processing two types of image sequences in parallel—skeleton heatmaps and RGB images. The 2S DR-AGCN solution employs graph structures besides the skeleton joints and branches. The top approaches analyze all the frames of a video clip, contrary to other methods, which process a sparse frame sequence only. Our results were obtained for 32 frames selected for windows of 64 frames.

### 4.4. Sliding Window Validation on a UT Subset

#### 4.4.1. The UT-Interaction Data Set

The models were also tested on the UT-Interaction data set [13], which contains longer videos with multiple interactions occurring one after the other. In total, five videos with eight interactions each were tested (the interactions were consistent with NTU classes). The accuracy of classification by our eight models is given in Table 4. The results confirm our findings based on the NTU RGB+D data set—the RAW features induce the worst classification accuracy, while the comparison of remaining models leads to the same ranking as before. The three best-performing models are TC-LSTM-LA, DC-LSTM-LA, and SC-LSTM-PS.

#### 4.4.2. Example of Multi-Interaction Video

**Figure 12:**
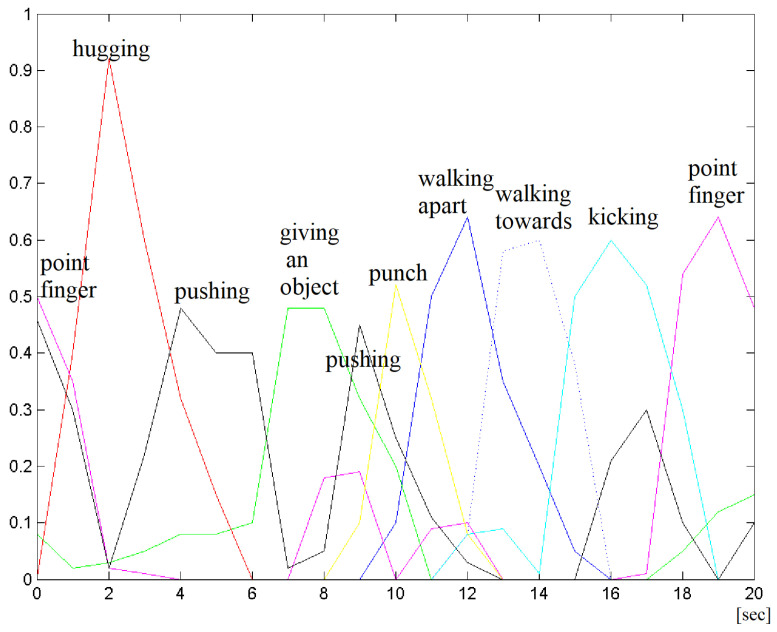
Illustration of interaction class likelihoods in true sliding window classification.

**Figure 13:**
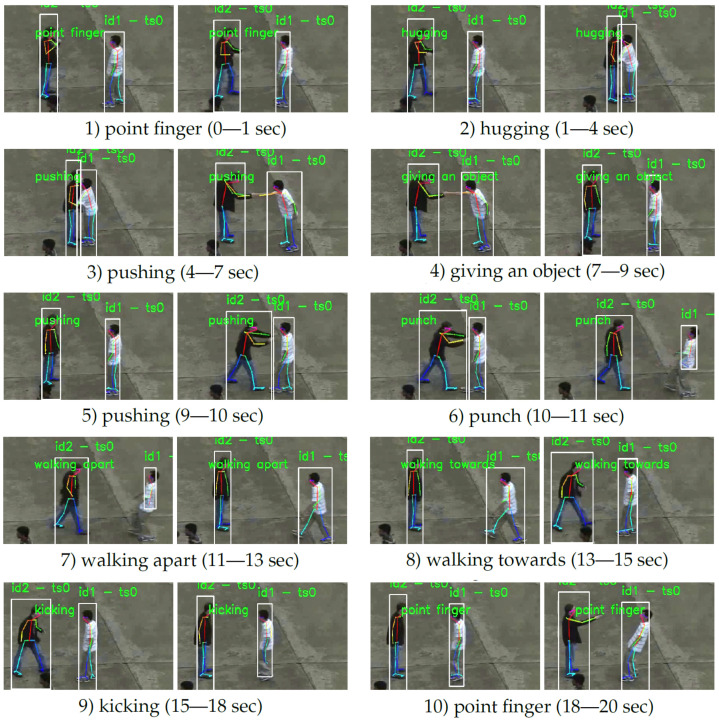
Illustration of detected interactions by sliding window classification.

Let us illustrate the strategy of sliding window classification on one example from the UT data set. The drawing in Figure 12 presents the development of interaction class likelihoods in the sequence of windows. For every window, the class with highest likelihood is chosen. The obtained results are collected in Table 5 and illustrated in Figure 13. The window size was 2 s, with interlace 0.5 (i.e., window rate was 1 window per second).

## 5. Discussion

As we can see from Table 3, many works on skeleton-based human activity recognition in video have been published in the last several years. They have been trained and evaluated on short video clips containing single activities. Our aim was to design an approach that solves a more realistic problem of processing a longer-time video with varying interactions between two actors. A second goal was to reach real-time processing with a satisfying classification performance. Our solution can be briefly characterized by three concepts: knowledge-aware skeleton feature extraction by the feature engineering step; use of multi-stream neural network models based on LSTM layers; and the sliding window-controlled processing of long-time videos.

We have trained several models on the interaction subset of the NTU RGB+D data set. The models have been evaluated in a short-video mode on the test part of the above training set and in a cross-model mode on long-videos from the UT-Interaction data set. The first evaluation resulted in the selection of the three best-performing single-, double- and triple-channel models: SC-LSTM-PS, DC-LSTM-LA, and TC-LSTM-LA. These models represent a tradeoff between accuracy and complexity, as the highest accuracy (of 94.9% when averaging the CV and CS scores) has been achieved by the most complex model TC-LSTM-LA (with 9.76 M weights), while the low complex model (with 3.33 M weights) showed the worst accuracy (of 92.75%). The usefulness of our feature engineering step can be verified by the presented results. When the raw skeleton data was used, the corresponding model has reached an average accuracy of only 77.9%.

A comparison with the top performing complex DNN models validated a good standing of our solutions. Our moderate complexity models with standard LSTM layers perform 3.4–5.55% lower than the currently best PoseConv3D(J+L) (with an average performance of 98.3%). Please note that this top version of the PoseConv3D family was trained not only on skeleton heatmaps but also on the original RGB data. The performance of our models is only slightly lower than the second-best performing adaptive graph convolutional networks (the 2S DR-AGCN model) with 95.93%.

Our models and the sliding window step have also been validated on a second data set—the UT-Interaction set of longer-time videos with many interactions. Again, the TC-LSTM-LA model has shown a highest accuracy of 97.5%. By monitoring the results obtained for consecutive window locations, one could also verify the almost perfect classification of multiple interactions (in the presented example—a proper classification of nine out of ten interactions).

The main scientific contribution is related to the proposed feature engineering algorithm that performs skeleton tracking and knowledge-aware (“hand-crafted”) relational feature extraction. This contribution can be formulated as follows:

1. We demonstrated the superiority of our approach—using hand-crafted relational features combined with an LSTM-based classification model over simple neural network models that learn relational features from pairs of joints—such as the IRN${}_{inter+intra}$ and LSTM-IRN models.
2. Our hand-crafted features can equalize the advantages of modern graph neural networks and graph convolutional networks over LSTMs, when both are applied in the feature transformation stage (as an encoder). Even complex configurations, such as the AS-GCN and 2S-GCN models, can be challenged by our approach.

## 6. Conclusions

An approach to two-person interaction classification has been designed and experimentally evaluated. The input data come from the OpenPose tool, which is an efficient deep network solution for generating human skeleton sets from an image or video frame. The quality of skeleton data is improved by the proposed skeleton tracking and joints correction procedure. An important quality contribution comes from the knowledge-aware feature engineering step, which generates relational data from the raw skeletons.

Various network configurations, based on LSTM layers, were trained and evaluated. High quality test results prove our concept. Applying our relational features, accuracy gains of 12–14% have been achieved compared to the use of RAW skeleton data. A practical advantage is the assumed sparsity of video frames. By adjusting the key frame number, real-time processing is possible even with moderate computational resources. The approach can easily be adopted to process true image sequences, such as image galleries.

The limitations of this study are as follows: a strong dependence on the proper estimation of human skeleton data by OpenPose or HRnet and a focus on main body parts, i.e., human actions performed by feet, hands, and fingers cannot be properly distinguished from each other.

## Figures and Tables

**Table 1:** The per-class test accuracy of three models trained on the NTU-RGB+D interaction set, verified in the CS (cross subject) mode.

	A050	A051	A052	A053
Model	Punch	Kicking	Pushing	Pat on Back
SC-LSTM-RAW	61.8%	80.7%	81.8%	63.5%
SC-LSTM-LA	79.2%	91.2%	91.3%	84.7%
TC-LSTM-LA	94.6%	94.4%	97.4%	95.2%
	**A054**	**A055**	**A056**	**A057**
**Model**	**Point Finger**	**Hugging**	**Giving Object**	**Touch Pocket**
SC-LSTM-RAW	64.0%	93.5%	64.2%	53.7%
SC-LSTM-LA	82.4%	97.1%	89.2%	80.4%
TC-LSTM-LA	95.0%	99.4%	95.6%	94.4%
	**A058**	**A059**	**A060**	
**Model**	**Shaking Hands**	**Walking Towards**	**Walking Apart**	
SC-LSTM-RAW	73.9%	98.5%	99.5%	
SC-LSTM-LA	88.4%	100%	99,6%	
TC-LSTM-LA	97.4%	100%	99.8%	

**Table 2:** The test accuracy of four models trained on the NTU-RGB+D interaction set, verified in the CS (cross subject) and CV (cross view) modes. The best CS- and CV-performances are highlighted by a bold font.

No.	Model	CS	CV	Parameters
1	SC-LSTM-RAW	75.7%	80.1%	3.33 M
2	SC-LSTM-LA	89.3%	90.5%	3.36 M
3	SC-LSTM-PD	91.7%	93.5%	4.12 M
4	**SC-LSTM-PS**	91.0%	**94.5%**	3.33 M
5	**DC-LSTM-LA**	95.5%	94.4%	6.61 M
6	DC-LSTM-PD	90.0%	91.8%	8.09 M
7	DC-LSTM-PS	90.1%	91.7%	6.53 M
8	**TC-LSTM-LA**	**96.6%**	93.2%	9.76 M

**Table 3:** Test accuracy of leading works evaluated on the NTU-RGB+D interaction set in the CS (cross subject) and CV (cross view) mode. Our three models are highlighted by a bold font.

Approach	Year	CS	CV	Parameters
FSNET [28]	2019	74.0%	80.5%	-
ST-LSTM [18]	2016	83.0%	87.3%	-
ST-GCN [21]	2018	83.3%	87.1%	3.1 M
IRN${}_{inter+intra}$ [3]	2019	85.4%	-	9.0 M
GCA-LSTM [29]	2017	85.9%	89%	-
2-stream GCA-LSTM [30]	2018	87.2%	-	-
AS-GCN [22]	2019	89.3%	93%	9.5 M
LSTM-IRN [3]	2019	90.5%	93.5%	9.08 M
2S-AGCN [23]	2019	93.4%	-	3.0 M
DR-GCN [25]	2021	93.6%	94.0%	3.18 M
2S DR-AGCN [25]	2021	94.68%	97.19%	3.57 M
PoseConv3D(J+L) [27]	2022	97.0%	99.6	6.9 M
SC-LSTM-RAW	2022	75.7%	80.1%	3.33 M
**SC-LSTM-PS**	2022	91.0%	94.5%	3.33 M
**DC-LSTM-LA**	2022	95.5%	94.4%	6.51 M
**TC-LSTM-LA**	2022	96.6%	93.2%	9.76 M

**Table 4:** Cross-domain test accuracy of our eight models obtained on the UT-Interaction data set.

No.	Model	Accuracy
1	SC-LSTM-RAW	72.5%
2	SC-LSTM-LA	82.5%
3	SC-LSTM-PD	90.0%
4	SC-LSTM-PS	92.5%
5	DC-LSTM-LA	95.0%
6	DC-LSTM-PD	87.5%
7	DC-LSTM-PS	90.0%
8	TC-LSTM-LA	97.5%

**Table 5:** Interactions detected in consecutive window periods of a 20 s video clip.

No.	First Frame	Last Frame	Detected Interaction	True/False
1	1	15	point finger	True
2	16	105	hugging	True
3	106	195	pushing	False (“walking apart”)
4	196	255	giving an object	True
5	256	285	pushing	True
6	286	315	punch	True
7	316	375	walking apart	True
8	376	435	walking towards	True
9	436	526	kicking	True
10	527	594	point finger	True

## Data Availability

Links to the data sets are included in the Reference section.
